# Changing Health Behaviors Using Annual Wellness Visits Among Senior Housing Residents

**DOI:** 10.1093/geroni/igaf122.3729

**Published:** 2025-12-31

**Authors:** Barbara Resnick, Nicole Brandt, Sarah Holmes, Jennifer Klinedinst, Anne Hagan

**Affiliations:** University of Maryland, Baltimore, Maryland, United States; UMB School of Pharmacy, 20 N. Pine St. Baltimore, Maryland, United States; University of Maryland School of Nursing, Baltimore, Maryland, United States; UMB SON, 655 W. Lombard Street, Baltimore, Maryland, United States; UMB SON, ELKRIDGE, Maryland, United States

## Abstract

More than 1.8 million older adults receive rental assistance in federally subsidized low-income housing communities. These individuals are mostly racial and ethnic minorities and experience multiple comorbid medical conditions, functional and cognitive impairment and have poor health behaviors. To help these individuals maintain or improve healthy behaviors we provided monthly interdisciplinary wellness clinics in four senior housing communities. Examples of services provided included blood pressure monitoring, cerumen removal, cognitive evaluations and management, nail and skin care, medication education and management, falls assessments and individualized exercise programs, among others. Annually, during the residents’ birthday month, they were invited to have an Annual Medicare Wellness Visit (AWV) which included setting health behavior goals. A sample of 105 residents had two Medicare Annual Wellness Visits. The mean age of the participants was 79(SD = 8), most were female (77%) and Black older adults (75%), with only 21% being Hispanic, 16% married, and 90% had at least some high school education. Changes over time were evaluated using general linear modeling for continuous variables and chi-square for dichotomous variables. There was no change in perceived health status, falls, help with activities of daily living, or alcohol use. There was a decrease in the percentage of residents that worried about falling, were smoking, not exercising, or did not have an advanced directive. Cognition declined over the two years based on the CLOCK test and the individual’s subjective memory complaints. Providing AWVs to senior housing residents may be helpful to maintain and improve healthy behaviors over time.

